# Larger Mid-Dorsolateral Prefrontal Gray Matter Volume in Young Binge Drinkers Revealed by Voxel-Based Morphometry

**DOI:** 10.1371/journal.pone.0096380

**Published:** 2014-05-02

**Authors:** Sonia Doallo, Fernando Cadaveira, Montserrat Corral, Nayara Mota, Eduardo López-Caneda, Socorro Rodríguez Holguín

**Affiliations:** Department of Clinical Psychology and Psychobiology, University of Santiago de Compostela, Santiago de Compostela, Spain; University of Jaén, Spain

## Abstract

Binge drinking or heavy episodic drinking is a high prevalent pattern of alcohol consumption among young people in several countries. Despite increasing evidence that binge drinking is associated with impairments in executive aspects of working memory (i.e. self-ordered working memory), processes known to depend on the mid-dorsolateral prefrontal cortex (Brodmann areas 46 and 9), less is known about the impact of binge drinking on prefrontal gray matter integrity. Here, we investigated the effects of binge drinking on gray matter volume of mid- dorsolateral prefrontal cortex in youths. We used voxel-based morphometry on the structural magnetic resonance images of subjects reporting a persistent (at least three years) binge drinking pattern of alcohol use (n = 11; age 22.43±1.03) and control subjects (n = 21; age 22.18±1.08) to measure differences in gray matter volume between both groups. In a region of interest analysis of the mid-dorsolateral prefrontal cortex, after co-varying for age and gender, we observed significantly larger gray matter volume in the left mid-dorsolateral prefrontal cortex (Brodmann areas 46 and 9) in binge drinkers in comparison with control subjects. Furthermore, there was a significant positive correlation between left mid-dorsolateral prefrontal cortex volume and Self-Ordered Pointing Test (SOPT) total errors score in binge drinkers. The left mid-dorsolateral prefrontal cortex volume also correlated with the quantity and speed of alcohol intake. These findings indicate that a repeated exposure to alcohol −that does not meet criteria for alcohol dependence− throughout post-adolescent years and young adulthood is linked with structural anomalies in mid-dorsolateral prefrontal regions critically involved in executive aspects of working memory.

## Introduction

Binge drinking (BD), or heavy episodic drinking, is characterized by repeated episodes of heavy alcohol consumption (leading to intoxication) followed by abstinence, and is now acknowledged as being the most common type of alcohol misuse among young people in several countries [1]. Despite the increased prevalence of this pattern of alcohol consumption during adolescence and early adulthood, the extent to which BD may affect brain integrity and cortical maturation has only been investigated recently. Of particular importance is how this pattern of repeated alcohol exposure affects the prefrontal cortex, one of the last brain regions to develop and that undergoes substantial developmental changes during this age span.

Studies of gray matter (GM) maturation show a loss in cortical GM density over time [2]–[3], which has been attributed to synaptic pruning and myelination, cellular changes known to occur throughout adolescence in humans [4]. Magnetic resonance imaging (MRI) studies have reported that, in the frontal lobe, the GM maturation ultimately involves the dorsolateral prefrontal cortex (DLPFC), which shows an increase in GM density reduction during the post-adolescent years [2]–[3]. In close parallel, significant improvements in high-order executive functions are observed at this stage of development [5].

Animal studies indicate that adolescence is a period of particular vulnerability to alcohol-induced neurotoxic effects. Intermittent administration of ethanol during adolescence induces prefrontal cortex damage [6]–[7].

In humans, alcohol-induced executive dysfunction and anomalous prefrontal functioning have been reported in adolescents and young people with Alcohol Use Disorder (AUD, defined as DSM-IV alcohol dependence or abuse), e.g. [8]–[11]. Regarding those studies in adolescents and young people reporting a BD pattern of alcohol consumption, alcohol-related functional abnormalities have been reported both in electrophysiological and hemodynamic correlates of cognitive function [12]–[18], including prefrontal-cortex dependent executive functioning [19]–[27]; for reviews see [1], [28]–[30]. At a neuropsychological level, binge drinkers show a variety of deficits on tasks assessing frontal executive function, such as attention and planning, cognitive flexibility, working memory, decision-making, word fluency, task switching and inhibitory control tasks [31]–[36]; for reviews see [1], [28]). Consistently, and importantly for the present study, in a recent work from our group [37], we found that adolescent binge drinkers performed worse than non-BD adolescents in tasks that depend on the integrity of the DLPFC. Specifically, BD resulted in poorer performance on tasks involving executive aspects of working memory (i.e. monitoring of information in working memory, such as self-generated responses) that have been consistently associated with activity in the mid-DLPFC (which comprises Brodmann's areas [BA] 46 and 9) [38]–[41]. These findings were in agreement with previous research by Scaife and Duka [34] also showing an impaired (gender-specific) performance in a spatial self-ordered task. Remarkably, we found these difficulties in self-ordered working memory to persist after maintenance of a binge-pattern of alcohol use over a 2-year period [42]. These results are also in line with other longitudinal studies showing a relationship between BD and altered executive functioning. For instance, prolonged BD has been associated with diminished decision-making [33]; furthermore, pre-existing altered activation in frontal regions has been observed in future alcohol-using adolescents during response inhibition [43] (see also [44]) and visual working memory [45].

Despite this increasing evidence for functional prefrontal impairments associated with BD, as well as for altered microstructural white matter integrity as measured by diffusion tensor imaging (DTI) [46]–[47], less is known about the impact of heavy episodic drinking on adolescents' prefrontal GM integrity. MRI studies in adolescents with AUD with [48] and without [49] comorbid psychiatric disorders have revealed smaller prefrontal total volume and prefrontal white matter volume in AUD adolescents compared to non-AUD adolescents. However, whether the observed abnormalities in total prefrontal volume among AUD adolescents were primarily due to white or gray matter pathology could not be established in these studies. Only very recently, one MRI study has provided evidence of differences (gender-specific) in cortical thickness in left frontal regions related to binge drinking in a non-clinical adolescent sample. Thicker frontal cortices were linked to poorer neuropsychological performance and were interpreted to represent altered pruning [50].

In this study, we used MRI and voxel-based morphometry (VBM) employing a region-of-interest (ROI) approach, to examine, based on our previous work, the effects of a persistent (at least three years) binge-pattern of alcohol use on GM volume of DLPFC (specifically, mid-DLPFC) in youths. We hypothesized that young binge drinkers would show structural abnormalities in mid-DLPFC compared with sociodemographically similar control subjects. Based on the scarce and inconclusive structural MRI literature available examining the effects of heavy alcohol use or abuse on GM integrity, we based our specific hypothesis about the direction of this effect in the work from Squeglia et al. [50]; i.e. we expected these alterations to be reflected as larger prefrontal volumes in binge drinkers relative to controls.

## Materials and Methods

### Participants

The neuroimaging assessment of participants was performed during their fourth academic year of attendance at the University of Santiago de Compostela, within the framework of a longitudinal study examining the neurocognitive consequences of BD on the young university population, and in which they had been assessed in their first [37] and third [42] academic year. Participants were contacted again and invited to participate in a MRI study. The 36 participants who agreed to participate were questioned about their current drinking pattern. Subjects were classified as binge drinkers (BD) if they reported drinking (i) six or more standard alcoholic drinks (10 g of alcohol according to the Spanish Health Authority's reference) on the same occasion at least once per week, or (ii) six or more standard alcoholic drinks on the same occasion at least once a month and, during these episodes, drank at a speed of consumption of at least three drinks per hour. They were classified as controls (CG) if they reported drinking six standard alcoholic drinks on the same occasion less than once per month and at a maximum speed of consumption of two drinks per hour. Participants had thus maintained these patterns (BD/control) of alcohol consumption for at least three years, according to our previous assessments. For sample consistency, four BD participants from the longitudinal study who did not report a current binge-drinking pattern of alcohol consumption were excluded from the analysis.

Sociodemographic and substance use data were collected through a questionnaire that included the Alcohol Use Disorders Identification Test (AUDIT)[51] and questions about consumption of other psychoactive substances (specific type of substance used, frequency of consumption, etc). The AUDIT has been validated to assess alcohol-related problems or disorders [51], and specifically in university students, for a review see [52]. The Galician validated version of the AUDIT [53] was used. Personal and family history of alcoholism and medical or psychopathological disorders information was collected through a semi-structured interview that comprised: a translated and adapted version of the Semi-Structured Assessment for the Genetics of Alcoholism (SSAGA), Individual Assessment Module (IAM) and Family History Assessment Module (FHAM), designed by the Collaborative Study on the Genetics of Alcoholism (COGA)[54]; and the Symptom Checklist-90-Revised (SCL-90-R)[55], which is a questionnaire for self-report of a range of current psychological symptoms and distress and measures nine primary symptom dimensions (interpersonal sensitivity, depression, anxiety, hostility, phobic anxiety, paranoid ideation and psychoticism).

The exclusion criteria were as follow: a score above 90 in the Global Severity Index (GSI) of the SCL-90-R(GSI is the average rating given to all 90 items and provides a measure of overall psychological distress) or in at least two of the symptomatic dimensions; personal history of neurological disorders; regular (i.e. on a weekly basis) consumption of cannabis or other drugs (legal or illegal) with psychoactive effects (whether prescribed or not); alcohol abuse/dependence according to the DSM-III-R criteria; personal and/or family history of major mental disorder and history of alcoholism in first-degree relatives; and MRI contraindications. Finally, 32 participants (mean age  = 22.34, SD = 1.04, range 20–24) were selected for neuroimaging assessment: 11 binge drinkers (7 males, 4 females) and 21 non-binge drinkers (10 males, 11 females). All subjects gave written informed consent after the procedure had been carefully explained and received 30 € for their participation.

### Ethics Statement

All participants were volunteers recruited from the University of Santiago de Compostela, and gave written consent to participate in this study for monetary compensation. The research was carried out in accordance with the ethical principles for research involving human subjects outlined in the Declaration of Helsinki, European Council Agreements and regulations on bioethics of Spain. The protocol was approved by the Bioethics Committee of the University of Santiago de Compostela.

### MRI Data Acquisition

Subjects were scanned with a Siemens 1.5 Tesla scanner (Siemens, Erlangen, Germany). High-resolution T1-weighted transversal images covering the whole brain were acquired with a magnetization-prepared rapid gradient echo (MP-RAGE) sequence: 192 slices with section thickness of 1.0 mm; flip angle  = 8°; echo time  = 4.38 ms; repetition time  = 2040 ms; field of view  = 192 mm. A neuroradiologist evaluated all scans for gross structural abnormalities. No participants were excluded.

### Image Processing

Structural images were processed with the voxel-based morphometry toolbox (VBM8) (http://dbm.neuro.uni-jena.de/vbm8/) implemented in Statistical Parametric Mapping (SPM8) (Wellcome Department of Imaging Neuroscience, London, UK) running on MATLAB 7.9.0 (R2009b; The Mathworks, Inc., Natick, MA, USA). VBM is a whole-brain, unbiased, semi-automated technique for characterizing regional cerebral differences in structural magnetic resonance images [56]. Segmentation of individual brains into gray matter, white matter, and cerebrospinal fluid was based on VBM8 using the default estimation options (i.e. very light bias regularization, 60 mm cut-off for estimating the Gaussian smoothness of bias in image intensity; ICBM [International Consortium for Brain Mapping] European template for initial affine transformation). Spatial normalization into the Montreal Neurological Institute (MNI) standard space was done by the high-dimensional DARTEL (Diffeomorphic Anatomical Registration Through Exponentiated Lie Algebra) approach implemented in VBM8. To this end, default options such as SANLM (spatial adaptive non local means) de-noising, light clean-up of partitions, and weighting of Hidden Markov-Random Fields by a factor of .15 were used. Subsequently, gray matter segments were modulated only by the nonlinear components to locally preserve actual GM values (so-called modulated volumes). Finally, the modulated volumes were smoothed with a 6-mm full-width-at-half-maximum (FWHM) Gaussian kernel. Normalized, modulated, smoothed images were submitted to group-level analyses.

### Statistical Analysis

To assess differences in regional gray matter volume between groups, two-sample t-tests analyses (CG >BD, CG <BD) were performed in SPM8. Age and gender were entered as covariates in all analyses. Because we had a strong a priori hypothesis targeting the mid-DLPFC, a region-of-interest (ROI) approach was used. These analyses were carried out using the Wake Forest University (WFU) Pickatlas toolbox (Version 2.5)[57] implemented in SPM8, with the left and right mid-DLPFC ROIs being anatomically defined as BA46 and BA9 in the Talairach Daemon [TD] Brodmann area atlas [58]. Although our primary focus was the mid-DLPFC because we had a strong hypothesis about a specific alteration of this region in BD, in order to additionally test whether there is a specific deficit among BD in the DLPFC or whether potential BD-related GM alterations could extend to other frontal regions (which are the brain regions more vulnerable to the neurotoxic effects of alcohol), further ROI analysis were performed on the following frontal areas: ventrolateral prefrontal cortex (BA44, BA45, BA47), orbitofrontal cortex (BA11, BA10), anterior cingulate cortex (BA24, BA32, BA33) and dorsolateral premotor cortex (BA6, BA8). To control for multiple statistical testing within our ROIs, a family-wise error (FWE) at *P*<0.05 was applied. For completeness of reporting, an exploratory whole-brain analysis was also carried out with a statistical threshold of *P*<0.001 (uncorrected for multiple comparisons) and minimum cluster size of 100 voxels.

We also examined the relationship between mid-dorsolateral prefrontal GM volumes and both neuropsychological executive performance and alcohol consumption variables. The neuropsychological data used for this study were collected as part of a study in which we reported poorer performance in young binge drinkers compared with control subjects in neuropsychological tests of executive functions that depend on DLPFC integrity [42]. Our main objective was to examine the relationship between mid-DLPFC volumes and the test in which significant differences in performance were found between BD and CG over the 2-year follow-up period (as reported in 37 and 42); namely, the Self-Ordered Pointing Test (SOPT)[59]. In the SOPT —abstract design version, 108 sheets are shown to the subject, each of which shows a series of abstract designs. This task is composed of four blocks with three trials each, in which the number of stimuli per sheet increases block by block (6, 8, 10 and 12). The stimuli are repeated on every sheet of each trial but the position changes at random from sheet to sheet. The task is to point to one design on each sheet, without repeating previously indicated designs. For each participant, the number of perseverative errors (errors that occurred as a result of pointing to the same item that was chosen on the immediately preceding sheet) and the total number of errors (the sum of perseverative and non-perseverative errors) are recorded. The SOPT assesses planning and self-monitoring aspects of working memory. In addition, in order to test whether there is a specific link between mid-DLPFC volume and performance on the SOPT or whether a relationship could be observed with other tests assessing executive functioning in our longitudinal study, further correlation analyses were performed between volumetric data and the scores in the Digit Span backward and the Spatial Location backward subtests of WAIS-III [60] that assess working memory, as well as in the Zoo Map and Key Search subtests of the Behavioral Assessment of Dysexecutive Syndrome (BADS)[61] that assess planning (details of these tests have been previously described, [37], [42]). Performance data on the neuropsychological tests were available for all the 32 subjects included in the present study (the average time elapsed between accomplishment of the neuropsychological assessment and acquisition of T1 images was from 0.7 to 2.4 years [1.8 years on average, SD = 0.63]). Participants were asked to abstain from consuming drugs and alcohol within 24 h prior to neuropsychological assessment. Table 1 contains descriptive demographic and neuropsychological data for the two groups. Differences between groups in frequency, quantity and intensity of alcohol consumption are also reported.

**Table 1:** Demographic, Neuropsychological and Drinking data (mean ± standard deviation) for Binge Drinking (BD) and Control samples.

	Control	BD
N (males/females)	21 (10/11)	11 (7/4)
Age (range)	22.43 (1.03)	22.18 (1.08)
White ethnicity (%)	100	100
Tobacco smokers	0	4a
Occasional use of cannabis	0	3b
SOPT total errors	7.29±4.29	9.45±4.85
Digits backward	5.24±1.38	5.64±1.36
Spatial location backward	5.86±1.2	6.45±0.82
Zoo Map, raw score	12.29±3.23	12.27±4.67
Key Search, raw score	11.62±3.49	12.55±2.50
Speed of consumption: drinks per hour*	0.62±0.57	2.23±0.88
Number of times consuming alcohol in the last week*	0.93±1.03	2.18±1.10
Grams of alcohol in a standard week*	29.90±35.46	225.91±57.22
Grams of alcohol in a BD day*	2.86±13.09	100.45±20.94
Grams of alcohol (estimated) within 2 hours of a BD episode*	1.43±6.55	67.73±26.21
Age of onset of alcohol use*	15.81±0.98	14.81±1.4

a<10 cigarettes per day (3 males, 1 female).

b<10 per month (one male BD participant consumed 10 cannabis cigarettes per month; one male and one female BD participants consumed one cannabis cigarette per month or less).

*t<0.05 significant group differences.

For the correlation analysis, the mid-DLPFC GM volumetric data were extracted from the coordinates of cluster maxima revealed by the ROI analyses using the MarsBar toolbox (v0.43) (http://marsbar.sourceforge.net/) for further analysis in PASW Statistics 18.0. Correlations between mid-dorsolateral prefrontal volumes and neuropsychological measures (scores on the tests) for the BD group were calculated by Pearson correlations. For BD and CG, GM mid-dorsolateral prefrontal volumes were also correlated with alcohol consumption variables measuring Speed of consumption (number of drinks consumed within one hour of a binge episode); Frequency (number of times consuming alcohol in a standard week); and Quantity (total grams of alcohol in a standard week; total grams of alcohol in a binge drinking day; estimated grams of alcohol within 2 hours of a binge drinking episode) as well as with the variable age of onset of alcohol use.

## Results

### Group Differences in Mid-Dorsolateral Prefrontal GM Volumes

#### BD > Control Subjects

**Figure 1:**
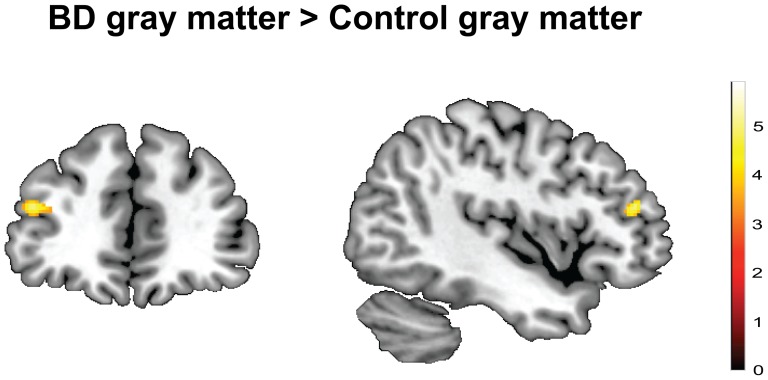
Group differences in gray matter volume. Color map showing larger gray matter volume in left mid-DLPFC (BA46 and BA9) in the binge drinking (BD) group compared with control subjects, after co-varying for age and gender (significant *P*<0.05 FWE-corrected at the voxel-level and at the cluster-level within regions of interest, and *P*<0.001 uncorrected on whole-brain analysis with minimum cluster size of 100 voxels). Color bar represents t-values.

The region-of-interest analysis revealed that the BD group had a greater GM volume of the left mid-DLPFC (BA46 and BA9) compared to the CG (MNI coordinates, −44, 42, 18; *t* = 5.10; *k* = 11; *P* = 0.012 FWE-corrected at the voxel-level; *P* = 0.017 FWE-corrected at the cluster-level)(Figure 1). No suprathreshold clusters were present for right mid-DLPFC ROI using the same thresholding criteria. The exploratory whole-brain analysis confirmed a significantly greater volume in a cluster in the left mid-DLPFC (MNI coordinates, −44, 42, 18; *t* = 5.10; *k* = 115; *P*<0.00l uncorrected)(Figure 1 and Table S1). At the exploratory whole-brain threshold, the BD group showed a greater GM volume in a number of brain regions, including occipital, limbic and frontal areas, compared to the control subjects (see Table S1 for details).

#### Control subjects > BD

Neither the ROI analysis, nor the whole brain analysis revealed any significant regions of increased GM in control subjects compared with binge drinkers.

### Additional ROI analysis of frontal regions

Additional ROI analysis of other anatomically defined frontal regions showed that in comparison to the CG, the BD group had greater GM volumes of the bilateral anterior cingulate cortex (ACC)(left: MNI coordinates, −2, 36, 24; *t* = 4.87; *k* = 8; *P* = 0.014 FWE-corrected at the voxel-level, *P* = 0.023 FWE-corrected at the cluster-level; right: 15, 2, 43; *t* = 5.88; *k* = 12; *P* = 0.001 FWE-corrected at the voxel-level, *P* = 0.018 FWE-corrected at the cluster-level); no significant differences were found in the ventrolateral prefrontal cortex, orbitofrontal cortex or dorsolateral premotor cortex. There were no significant regions of increased GM in CG relative to BD group.

### Correlation between Mid-Dorsolateral Prefrontal GM Volume and Executive Neuropsychological Performance

The correlation analyses for the BD subjects indicated that the left mid-DLPFC (BA46 and BA9) volume was positively correlated with SOPT total errors score (*r* = 0.558, *P* = 0.037). The same analysis for the control group was no significant (*r* = 0.092, *P* = 0.346). There were no significant correlations between the left mid-DLPFC volume and the other neuropsychological tests assessing executive functioning, neither in the BD group (Zoo Map, direct score: *r* = −0.226, *P* = 0.252; Key Search, direct score: *r* = −0.434, *P* = 0.091; Digit Span backward: *r* = −0.316, *P* = 0.172; Spatial Location backward: *r* = −0.414, *P* = 0.103) nor in the CG (Zoo Map, direct score: *r* = −0.125, *P* = 0.295; Key Search, direct score: *r* = −0.169, *P* = 0.232; Digit Span backward: *r* = 0.291, *P* = 0.100; Spatial Location backward: *r* = 0.082, *P* = 0.361).

### Correlation between Mid-Dorsolateral Prefrontal GM Volume and Pattern of Alcohol Consumption

Significant positive correlations between the left mid-DLPFC volume and variables measuring Speed (number of drinks consumed within one hour of a binge episode, *r* = 0.323, *P* = 0.036) and Quantity of consumption (total grams of alcohol in a standard week, *r* = 0.418, *P* = 0.009; total grams of alcohol in a binge drinking day, *r* = 0.472, *P* = 0.003; estimated grams of alcohol within 2 hours of a binge drinking episode, *r* = 0.450, *P* = 0.005) were observed across groups. A significant negative correlation was also found with the variable age of onset of alcohol use (*r* = −0.486, *P* = 0.005). There was no correlation between left mid-DLPFC volume and the variable measuring frequency of alcohol use (i.e. number of times consuming alcohol in a standard week; *r* = 0.118, *P* = 0.261).

### BD without regular consumption of cannabis

To be sure that the difference in cannabis consumption between groups was not confounding the results, we re-analyzed the data excluding one male BD participant who reported a consumption of cannabis that could be considered as more regular (i.e. 10 cannabis cigarrettes per month).

The results confirmed the larger volume of the left mid-DLPFC [BA46 and BA9) in the BD group relative to the CG (−45, 42, 18; *t* = 5.32, *k* = 17, *P* = 0.008 FWE-corrected at the voxel-level, *P* = 0.012 FWE-corrected at the cluster-level) and the significant positive correlations between left mid-DLPFC volume and (i) SOPT total errors score (*r* = 0.692, *P* = 0.013) and (ii) consumption variables measuring Speed of consumption (number of drinks consumed within one hour of a binge episode, *r* = 0.322, *P* = 0.039) and Quantity of consumption (total grams of alcohol in a standard week, *r* = 0.441, *P* = 0.007; total grams of alcohol in a binge drinking day, *r* = 0.477, *P* = 0.003; estimated grams of alcohol within 2 hours of a binge drinking episode, *r* = 0.453, *P* = 0.005); again, no correlations were observed with frequency of consumption. The significant negative correlation of left mid-DLPFC volume with age of onset of alcohol use was also corroborated (*r* = −0.492, *P* = 0.005).

The additional ROI analysis of other anatomically defined frontal regions showed again greater GM volumes of the bilateral ACC in BD relative to CG (left: MNI coordinates, −2, 36, 24; *t* = 4.61; *k* = 3; *P* = 0.027 FWE-corrected at the voxel-level, *P* = 0.033 FWE-corrected at the cluster-level; right: 15, 3, 43; *t* = 6.40; *k* = 20; *P* = 0.0005 FWE-corrected at the voxel-level, *P* = 0.012 FWE-corrected at the cluster-level).

The correlation analysis between the left mid-DLPFC volume and the other neuropsychological tests assessing executive functioning did not show either any significant effect for the BD group.

## Discussion

The major finding of the present study is that young university students reporting a binge drinking pattern of alcohol consumption, with a history of at least three years of binge drinking (and who started alcohol consumption during adolescence), had a significantly larger GM volume of left mid-DLPFC (BA46 and BA9) than age-matched non-binge drinkers students. Furthermore, our results indicate that these cortical volume differences in GM were positively associated with poorer performance in neurocognitive measures of executive functioning (i.e. monitoring of information in working memory) that are known to depend on the integrity of the mid-DLPFC, as well as with the quantity and intensity of alcohol intake.

Our results are in line with a recent study indicating that repeated binge drinking during adolescence is associated with differences in left frontal cortical thickness [50], and proposing a relationship between thicker frontal cortices and less neurodevelopment. MRI studies have revealed a robust increase in local GM density loss in the dorsal frontal regions that control executive functioning between adolescence and adulthood [2], being one of the last brain regions to develop fully [3]. These post-adolescent changes in GM density have been attributed to both regressive (i.e. synaptic pruning) and progressive (i.e. myelination) cellular changes known to occur simultaneously in the brain during childhood, adolescence and young adulthood, and considered indices of neural maturation and enhanced neural processing efficiency [2]–[4]. Although care should be taken in interpreting our present results (it is possible that the differences in brain structure were premorbid to BD and predisposed subjects to heavy episodic alcohol use), the larger GM mid-DLPFC volume associated with BD might reflect the deleterious effects of heavy episodic alcohol exposure on typical development of prefrontal cortex, a region particularly sensitive to alcohol neurotoxicity [62]. Support for this hypothesis comes from the following several findings. Firstly, the positive relationship between the left mid-DLPFC volume and quantity and speed of alcohol intake, variables that actually define a risky binge drinking behavior (i.e. the high quantity and the speed at which alcohol is consumed during a single session). Secondly, the negative relationship between the left mid-DLPFC volume and age of onset of alcohol use, suggesting that as earlier the onset of consumption during adolescence the larger the volume in comparison to non-binge drinkers youths. Thirdly, other frontal anatomically defined regions of interest showed a similar pattern of volume alterations, namely the bilateral ACC. The ACC has been proposed to form part of a cognitive-control network (together with the lateral prefrontal and parietal cortices) subserving regulatory control over impulsive and risky behavior [63] and has been included in neural models of addiction, e.g. [64]–[65]. Structural GM abnormalities involving the ACC have been reported in drug abuser populations, such as cocaine-dependent subjects [66]; interestingly, work by Cheetham et al. [67] indicates that pre-existing individual differences in structural morphology (i.e. smaller volumes) of the left dorsal and rostral paralimbic ACC in adolescents (age 12) predicted problematic alcohol use four years later (age 16).

Finally, to highlight that a similar interpretation has been proposed very recently in a VBM study [68] examining subcortical GM differences (in ventral striatum, hippocampus and amygdala) related to BD in college-aged young adults, which found larger bilateral ventral striatum volumes in binge drinkers compared to controls. Based on evidence indicating that striatal volumes decline in GM between adolescence and young adulthood, the authors interpreted the enlarged volumes in terms of relative neuroanatomical inmaturity in this population.

Despite these convergent observations from the present and previous studies, further longitudinal VBM studies examining the effects of a binge pattern of alcohol use from adolescence to young adulthood are warranted to confirm this hypothesis.

The finding that the increase in volume in left mid-DLPFC was positively associated with poorer performance in the SOPT may also provide support for this hypothesis. The SOPT assesses executive aspects of working memory, specifically the ability of monitoring a sequence of responses in working memory. Importantly, lesion studies in non-human primates [40], [69] and functional neuroimaging studies [38], [41] have shown that successful performance of this task is associated with activity in the mid-DLPFC (BA46 and BA9). Based on this well-established association, hypothesis-driven correlation analysis between GM volume in mid-DLPFC and the performance on the SOPT was conducted. In agreement with this evidence, our results indicate that the larger mid-DLPFC volume of binge drinkers, which could be considered as an index of less neural processing efficiency in this region, was linked to less efficient executive functioning as measured by the SOPT. However, to interpret the present results, the following limitation should be acknowledged: all participants of this cross-sectional neuroimaging study came from a longitudinal study by our group examining the neurocognitive consequences of BD on the young university population, in which students had been assessed during a follow-up period of 2-years [37], [42], and which revealed that maintenance of a binge-drinking pattern was associated with poorer performance in the SOPT at both assessment times. Given that the neuropsychological data used in this study was taken from the assessment closer in time to MRI data acquisition (i.e. second evaluation time, see [42]), the time interval between scan and neuropsychological data collection could span from 0.7 to 2.4 years. Acknowledging the limitation of comparing structural and cognitive measures collected with such time lapse between them, we focused our main analysis on the one test in which performance has been consistently associated to mid-DLPFC (BA46 and BA9) functioning and in which performance impairments have been repeatedly found at different follow-ups in our sample (thus suggesting the high sensitivity of the SOPT to BD-related alterations). In line with the expected specific link between mid-DLPFC volume and SOPT performance, no significant correlations were found between volume of this brain region and performance in others tests assessing executive function in our sample. Nevertheless, because neural maturation is still expected to continue over that time period, caution should be taken when interpreting these results.

Taken together, the present neuroimaging findings extend our previous neuropsychological work by revealing that a repeated exposure to alcohol throughout post-adolescent years and young adulthood is linked with structural anomalies in mid-dorsolateral prefrontal regions critically involved in executive aspects of working memory (i.e. monitoring of information within working memory)[70]. Our results are also consistent with recent studies [36] showing that heavy social drinking adversely affects performance on random letter generation tasks, which engage working memory and executive processes (i.e. holding information ‘on line’, suppression of habitual response patterns, internally driven response generation and monitoring of responses) and which has been shown to be linked to the left dorsolateral prefrontal cortex [71].

Several limitations of this study warrant consideration. First, recruitment of subjects was limited to students who participated in a five-year longitudinal follow-up study and, within the binge-drinking group, to those who maintained this pattern of alcohol use over time. The selection criteria resulted in a small, unequal, sample size, thus rendering our analysis susceptible to a type-II error and low statistical power to detect differences between the groups (although effects were indeed observed). Additionally, the unequal number of males and females within and between groups precluded us from exploring the influence of gender, which has been shown to moderate the relationship between structural abnormalities and alcohol consumption (e.g. [49], [50]). Future studies with larger, even sample sizes will be necessary to confirm the present results. A second limitation already mentioned is that the cross-sectional nature of this study makes it difficult to draw conclusions about causal relationships between larger mid-DLPFC gray matter volumes and BD. Longitudinal studies are necessary to examine if larger mid-DLPFC volume represents a vulnerability to, or a consequence of, heavy episodic drinking during adolescence.

Despite these limitations, we were still able to demonstrate reliable structural differences between a BD group and control subjects in the mid-DLPFC (BA46 and BA9), a brain region critically involved in high-level executive control. Our work has thus significant implications for understanding the potential deleterious consequences of a binge pattern of alcohol consumption − that does not meet criteria for alcohol dependence − on the neurodevelopment of prefrontal regions during the post-adolescent years.

## Supporting Information

Table S1 **Regions of increased gray matter volume in Binge Drinking (BD) group in comparison with Control group revealed by a whole-brain analysis conducted at p<0.001, uncorrected, and a minimum cluster size of 100 voxels.**
(DOC)
