# Supplementary material for: Larger Mid-Dorsolateral Prefrontal Gray Matter Volume in Young Binge Drinkers Revealed by Voxel-Based Morphometry
Source: PLoS One. 2014 May 2;9(5):e96380. doi: 10.1371/journal.pone.0096380 (PMC4008532; doi:10.1371/journal.pone.0096380)
Supplement: Table S1 — Regions of increased gray matter volume in Binge Drinking (BD) group in comparison with Control group revealed by a whole-brain analysis conducted at p<0.001, uncorrected, and a minimum cluster size of 100 voxels. (DOC) [file pone.0096380.s001.doc]

| Region/BA | L/R | MNI coordinates (mm) | | | Cluster size (nº voxels) | z | t |
| --- | --- | --- | --- | --- | --- | --- | --- |
|  |  | x | y | z |  |  |  |
| Middle Cingulate gyrus (BA24) | R  R | 18 | -16 | 42 | 240 | 4.47 | 5.46 |
|  |  | 12 | -12 | 34 |  | 3.92 | 4.57 |
| Middle Occipital Gyrus  (BA 19) | R  R | 34 | -93 | 15 | 118 | 4.27 | 5.12 |
|  |  | 38 | -94 | 6 |  | 3.36 | 3.77 |
| Middle Frontal Gyrus (BA46) | L | -44 | 42 | 18 | 115 | 4.25 | 5.10 |
| Anterior Cingulate /Medial Frontal Gyrus (BA32) | L  R | -2 | 36 | 24 | 190 | 4.11 | 4.87 |
|  |  | 14 | 36 | 33 |  | 3.94 | 4.60 |
| Precentral Gyrus/Middle Frontal Gyrus (BA6) | L  L | -50 | 3 | 42 | 101 | 3.93 | 4.59 |
|  |  | -58 | -1 | 42 |  | 3.53 | 4.00 |

BA, Brodmann Area; L, left; R, right
